# Development of a new suction instrument to reduce surgical smoke exposure in the operating room

**DOI:** 10.3389/fmedt.2026.1796828

**Published:** 2026-07-10

**Authors:** Weiwei Chen, Lumin Lei, Mingzi Xiao, Qiaoling Chen, Yanlan Ma

**Affiliations:** 1 Department of Urology, Fourth Medical Center, Chinese PLA General Hospital Beijing, China; 2 Department of Anesthesiology, the Fourth Medical Center, Chinese PLA General Hospital, Beijing, China; 3 Nursing Department, Hainan Hospital of PLA General Hospital, Sanya, Hainan Province, China; 4 Medical Service Training Center, Chinese PLA General Hospital, Beijing, China

**Keywords:** operation room, PM2.5, suction tube, surgical smoke, VOCs

## Abstract

**Objective:**

To remove surgical smoke and its toxic components, we developed a novel smoke removal tube nested with the intraoperative suction tube, enabling convenient application during surgery and reducing hazards of surgical smoke exposure to medical staff.

**Methods:**

Standardized cuts with an electric knife were performed on pig dorsal tissue in an animal laboratory operating room without vertical laminar flow. The generated surgical fumes were analyzed using microcomputer laser dust meter counters for real-time monitoring of particulate matter 2.5 μm (PM2.5). Three new design suction tubes with different orifice diameters (1.5, 2.0, and 2.5 mm), each connected to local smoke evacuation, were compared with a conventional instrument without smoke evacuation.

**Results:**

The PM2.5 levels were significantly reduced with all three new tools, with smoke reduction rates of 24.20%, 66.86%, and 43.02%, respectively. Ten seconds after cutting, the 2.0-mm-diameter suction tube demonstrated lower PM2.5 concentrations compared with the other two groups, and the difference was statistically significant (*P* < 0.05).

**Conclusion:**

This study is the first to develop an “all-in-one” surgical smoke device that enables simultaneous suction of smoke and blood with one hand. Among the tested tools, the 2.0-mm-diameter instrument is the best one. These results promote the use of local smoke evacuation and may inspire other surgeons to further innovation in this field.

## Introduction

Surgical smoke, a visible and hazardous by-product, is produced by heat-generating surgical instruments (1). Numerous studies have shown that surgical smoke contains particulate matter and volatile organic compounds, such as acetonitrile, benzene, hydrogen cyanide, and toluene (2–4). These hazardous substances can cause respiratory disease and affect the nervous and cardiovascular systems. In particular, benzene is carcinogenic and mutagenic, increasing the risk of bone marrow failure and depression (5). Surgical smoke may pose risks to the health of medical personnel through bacteria and viruses—a serious issue during the COVID-19 pandemic (6, 7). One study reported that the average daily amount of surgical smoke produced was equivalent in mutagenicity to approximately 27–30 cigarettes (8). Traditionally, suction tubes have been primarily used to remove waste liquid from surgical incisions, and only partially suction surgical smoke. Given the small pores of traditional suction tubes, when a large amount of smoke is generated, it is difficult to suction it quickly (9, 10). The Occupational Safety and Health Administration reported that approximately 500,000 surgeons, nurses, anesthesiologists, and technicians in America are exposed to surgical smoke each year (11).

To reduce these potential hazards, national institutes for occupational safety and health strongly recommend the use of mobile or portable smoke evacuation systems (12). Recent studies have reported the efficacy of such instruments in significantly reducing surgical smoke (13–15). Liu et al. (14) found that a para-incisional smoke evacuator reduced average smoke levels by 59.7% in spinal surgery, while Seipp et al. (15) reported that electrosurgical knives achieved a smoke capture efficiency of 91%. However, local surgical evacuation is not widely used across the world. In Switzerland, only 10% of surgical units have SESs (12). In China, Sun et al. (16) reported that only 23.1% of hospitals used local surgical smoke evacuation. Insufficient awareness of the risks posed by surgical smoke among surgeons constitutes the primary barrier to the popularization of surgical smoke evacuation systems. Michaelis et al. (17) conducted an online survey in 2018, which yielded a surgeon response rate of 5%, indicating a major lack of interest and knowledge regarding surgical smoke hazards. In addition, several researchers have reported that the costs of disposable instruments have limited their widespread dissemination in operating rooms worldwide (18, 19). Seipp et al. (15) further reported that SEDs generate noise levels between 51 and 69 dB, exceeding recommended threshold limits of 60 dB or less. In this study, to address the identified shortcomings, we designed a new suction instrument with three variations. This device enables surgical assistants to suction both blood and smoke at the same time with one hand. This study aims to select the best design by examining the utility of the three new suction tubes in reducing surgical smoke exposure in an animal laboratory operating room.

## Materials and methods

### Development of the new suction instrument

#### Device design

**Figure 1:**
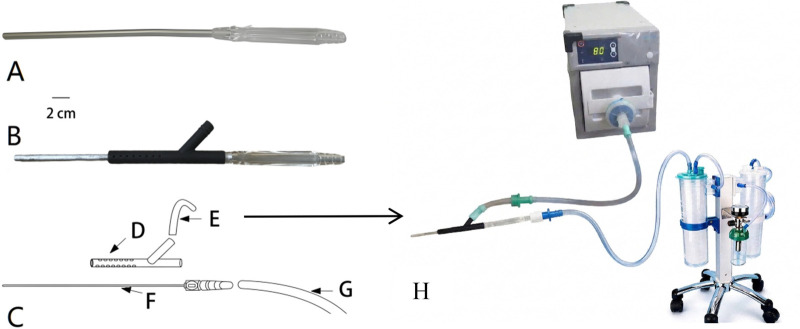
**(A)** Conventional suction tube; **(B)** new suction instrument as viewed from above; **(C)** connection diagram of the new device; (**D**) connection to local smoke evacuation system; (**E**) smoke removal pipe; (**F**) traditional suction tube; (**G**) connection to central vacuum aspiration system; (**H**) full system schematic.

To reduce interference for the surgeon, the new tool was designed with a smaller diameter and intended to accomplish both smoke evacuation and blood suction. The authors discussed the design scheme with surgeons and worked collaboratively with Tuoren Medical Device Company (He Nan, China). In order to increase the area for smoke removal and connect with the local smoke evacuation system, the suction tube was placed outside the conventional suction tube typically used by assistants (Figure 1).

**Figure 2:**
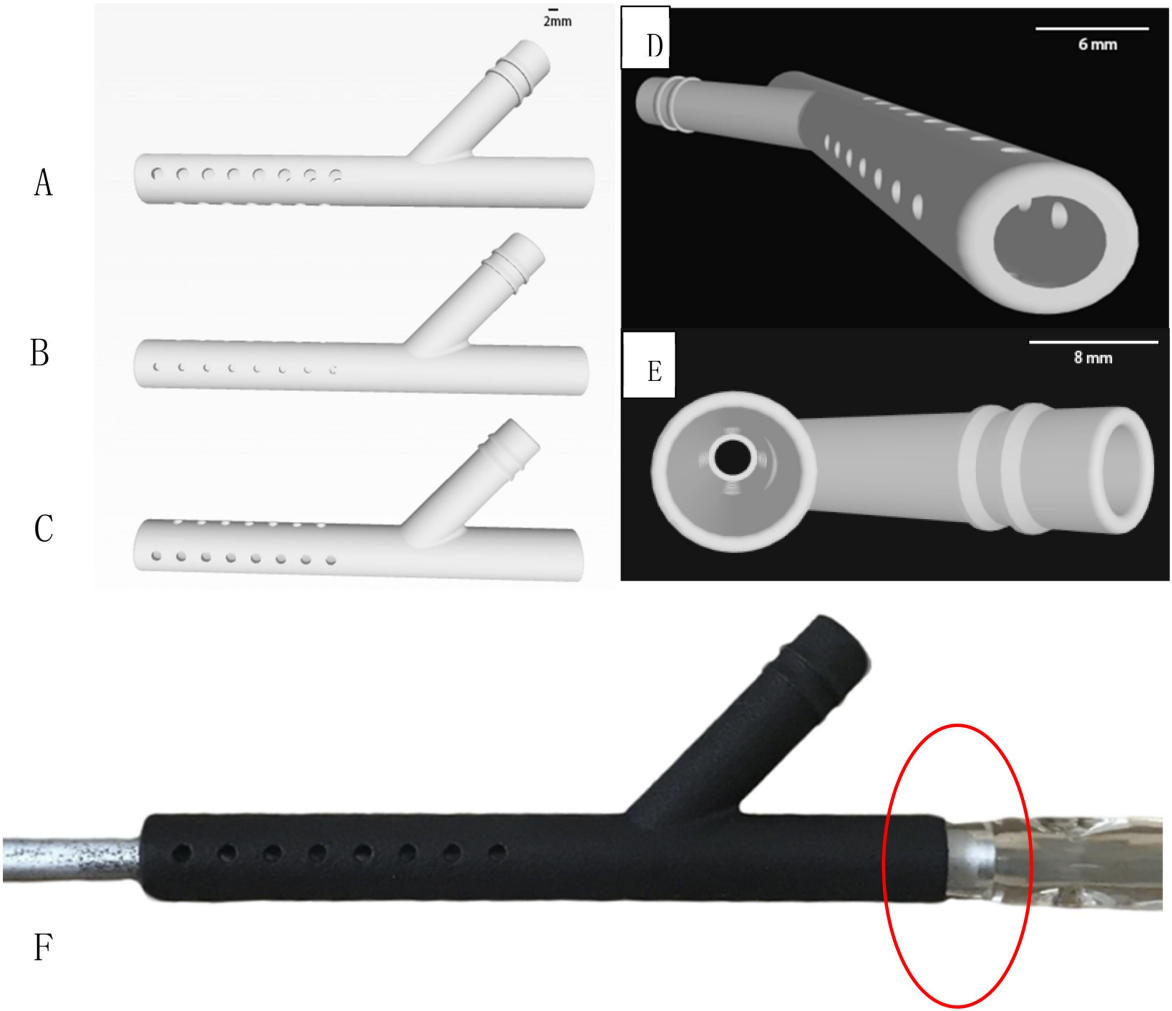
**(A)** Scheme with a 2.5 mm side-wall opening; **(B)** scheme with a 1.5 mm side-wall opening; **(C)** scheme with a 2.0 mm side-wall opening; **(D)** front of the outer tube; **(E)** end of the tube; **(F)** connection details, with the red circle indicating the outer tube nested onto the inner tube.

The inner tube serves as a disposable suction device for open surgeries, as illustrated in Figure 1. It measures 20 cm in total length and has an outer diameter of 4 mm, featuring a deformable lumen shape. The outer tube is designed in a “ￛ” configuration. One end of its tail is integrated into the suction tube handle, while its side interface connects to the smoke exhaust device, as depicted in Figure 1. The front end is designed with a semi-closed opening with a diameter of 6 mm (Figure 2). To facilitate placement of the suction tube in narrow and elongated surgical fields for removal of accumulated blood and fluids without interfering with the operation, a distance of 9 cm was established between the front end of the outer tube and the front end of the inner tube. Consequently, the total length of the outer tube is 9 cm, with an inner diameter of 8 mm and an outer diameter of 9 mm. The wall thickness is 0.5 mm, and the wall features staggered perforations. Initially, 30 holes were incorporated into the tube wall, designed with three different hole diameters of 1.5, 2, and 2.5 mm, as summarized in Table 1, to identify the design that achieved optimal smoke removal. Three new suction tubes with different orifice diameters (1.5, 2, and 2.5 mm) were designed, so as to achieve maximum smoke removal.

**Table 1:** Design scheme for external pipe wall opening.

Scheme	Diameter of the hole (mm)	Longitudinal hole spacing (mm)	Seven hole columns	Eight hole columns	Total number of holes	Total area of openings (mm^2^)
1	1.5	5.5	2	2	30	53.0
2	2.0	5.0	2	2	30	94.2
3	2.5	4.0	2	2	30	147.2

The research team has previously conducted simulation analysis using COMSOL Multiphysics. Among the three designs, the smoke extraction pipe with a hole diameter of 1.5 mm had the highest flow velocity, while the pipe with a hole diameter of 2.5 mm had the lowest flow velocity. Based on flow velocity and pressure at the interface, the flow at the outlet of the three designs was calculated using surface integration. The flow rates for hole diameters of 1.5, 2, and 2.5 mm were 0.00007, 0.00008, and 0.00009 m^3^/s, respectively. The smoke extraction pipe with a hole diameter of 2.5 mm had the highest flow rate (20). Finally, CAD software was used to design the drawing, and the pipe fittings were printed using 3D technology.

#### Enrollment and experimental setup

**Figure 3:**
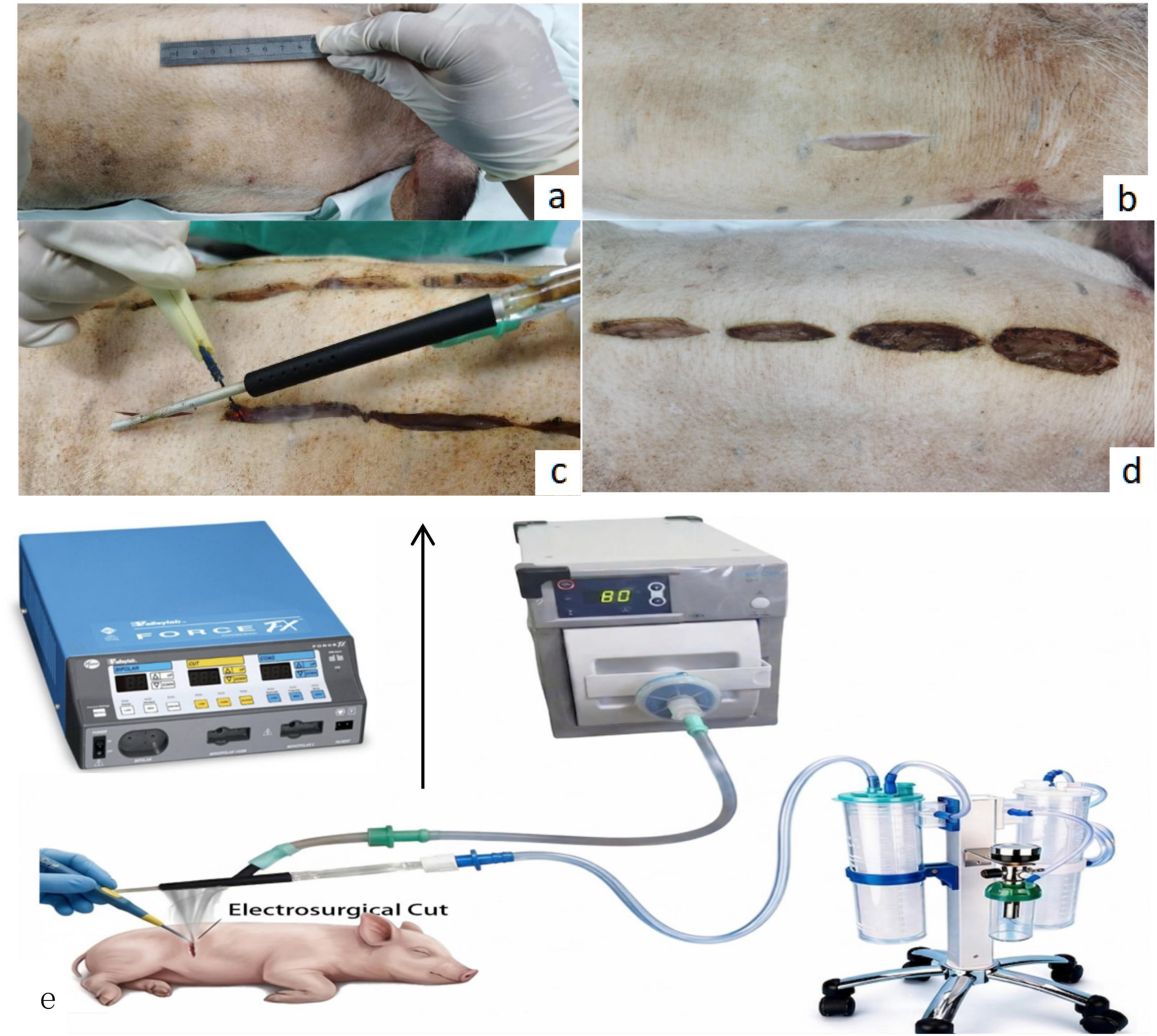
**(a)** Numbered at 5 cm intervals; **(b)** scalpel cutting a 4-cm-long incision; **(c)** electrocautery cutting for 20 s with visible smoke; **(d)** cutting completed; **(e)** complete experimental setup.

Approved by the ethical review system for animal experiments of the hospital (No. 2022-X18-70), this study was conducted in the Experimental Animal Surgery Laboratory of the PLA General Hospital. The temperature in the animal operating room was 25 °C, the relative humidity was 58%, and the air change rate was 8–15 times/h. Electrosurgical smoke was produced by applying electrocoagulation to the back of a Bama miniature pig in an operation theater without a laminar airflow system (Figure 3). The Bama miniature pig was fasted 12 h before surgery and injected with atropine 0.05 mg/kg, midazolam 0.1 mg/kg, and morphine hydrochloride 5 mg 30 min before surgery. The limbs of the animal were fixed in the supine position, intravenous channels were established after sedation, and overdose (>150 mg/kg) was injected intravenously, resulting in cardiac arrest. Surgical smoke was generated using conventional electrocautery (Force FX, Valley Lab, Boulder, CO) in coagulate mode at an electrosurgical power setting of 60 W. This setting was chosen because the “cut mode” has been reported to be effective between 50 and 80 W, allowing sufficient cutting through muscle tissue even when eschar fouled the stainless-steel blade electrode (14, 21). These settings are the commonly used in the OR at our spinal surgical unit. The three new suction tubes and one conventional tube were tested.

This experiment involved 32 experimental sites. Along the cephalocaudal axis of the dorsal spine of one miniature Bama pig, a total of 32 incision regions were demarcated with each segment spanning 5 cm. The regions were randomly assigned to four groups (8 regions per group), namely the conventional suction tube group, 1.5 mm diameter group, 2 mm diameter group and 2.5 mm diameter group. Cuts of a defined length of 4 cm were performed for a defined period time of 20 s (Figure 3). The suction tubes of the corresponding group were used to remove the surgical smoke, with all the new suction tubes fixed at the root of the electric knife pen. In between the cuttings, a break of 10–20 min was taken until the microcomputer laser dust meter counters showed that the particulate matter 2.5 μm (PM2.5) was back to normal levels (as measured before the beginning of the experiments). All procedures were performed by the same surgeons.

#### Smoke evacuation instruments

The three newly designed suction tubes were used in the experimental groups, while the conventional device was used as the control. The new suction instrument was connected to the surgical smoke evacuation system IES2 (ERBE Swiss AG, Winterthur, Switzerland), which was operated using a pedal switch. The suction power was >550 L/min. The new device had an “all-in-one” design, where a smoke evacuation cannula was assembled as part of and the conventional suction tube, which is used for blood removal. When the surgical pencil produced smoke while cutting the pig tissue, evacuation was activated using the pedal switch at 60% suction power, which was used in previous studies (14–16). The smoke was captured by the new suction tube and carried to the evacuation filter via a suction hose, while the conventional suction device collected fluid. In the control group, a traditional disposable instrument was used for both smoke removal and fluid collection.

#### Microcomputer laser dust meter counters LD-5C

The level of smoke exposure during the experiment was monitored in real time using the LD-5C microcomputer laser dust meter counter (JuChuang HuanBao Technology, Qingdao, China). The measurement range of the device extends from 0.001 to 10 mg/m^3^, with a detection sensitivity of 0.001 mg/m^3^. It operates at a sampling flow rate of 2 L/min and can be equipped with filters for PM10, PM5, and PM2.5 particles, thereby fulfilling the monitoring requirements for various particle sizes, which was set at 2.5 μm for this study. The LD-5C is a rapid dust-measuring instrument with laser as light source, which can directly read the dust mass concentration and is suitable for rapid determination of indoor inhalable particle concentration (22).

#### Study items

The temperature, humidity, and air exchange rate in the room were recorded for each experiment. The inlet of one dust meter was placed 40 cm above the meat pieces, with another dust meter placed 2 m away from the surgeon as a control. The distance of 40  cm directly above the incision site corresponded to the surgeon's breathing zone, while the distance of 2 m aligned with the circulating nurse's breathing zone. A total of 8 measurements of ambient PM2.5 concentration were performed at the following time points: prior to electrosurgical cutting, 5, 10, 15 and 20 s after cutting commenced, and 5, 10 and 15 s upon termination of cutting. The concentrations of 2.5 μm particulate matter for the four groups were analyzed and at different detection times for each group. The average smoke level was calculated as the “cumulative time-average concentration” of the smoke particles during the surgery (12), representing the mean of different detection times of the four groups.

### Statistical analysis

Data were analyzed using SPSS26.0. Results are reported as mean ± standard deviation for each experiment. Analysis of variance was used to compare the level of smoke exposure among the four groups (three new suction tube groups and one conventional control group). The *P*-value was set at 0.05.

## Results

### Concentration of 2.5 μm particulate matter in four groups

For the traditional suction tube without local smoke evacuation, the average smoke level measured was 394.11 ± 209.34 μg/m^3^. The average smoke levels for the new suction tubes with orifice diameters of 1.5, 2.0, and 2.5 mm, when the smoke evacuator was activated, were 318.67 ± 248.42 μg/m^3^, 130.59 ± 120.22 μg/m^3^, 224.56 ± 207.71 μg/m^3^, respectively. The PM2.5 concentration with the 2.0 mm orifice diameter was significantly lower than that of the conventional device (*P* < 0.05, Table 2). Pairwise comparisons among the remaining groups showed no statistically significant differences.

**Table 2:** Pairwise comparison of PM2.5 concentration among four groups.

Groups		Mean deviation	Standard error	*P-*value
Traditional suction tube	Diameter 1.5 mm	75.43	100.98	0.461
	Diameter 2.0 mm	263.51	100.98	0.014
	Diameter 2.5 mm	169.54	100.98	0.104
Diameter 1.5 mm	Traditional suction tube	−75.43	100.98	0.461
	Diameter 2.0 mm	188.07	100.98	0.073
	Diameter 2.5 mm	94.10	100.98	0.359
Diameter 2.0 mm	Traditional suction tube	−263.51	100.98	0.014
	Diameter 1.5 mm	−188.07	100.98	0.073
	Diameter 2.5 mm	−93.96	100.98	0.360
Diameter 2.5 mm	Traditional suction tube	−169.54	100.98	0.104
	Diameter 1.5 mm	−94.10	100.98	0.359
	Diameter 2.0 mm	93.96	100.98	0.360

### Concentration of 2.5 μm particulate matter at different detection times across four groups

**Figure 4:**
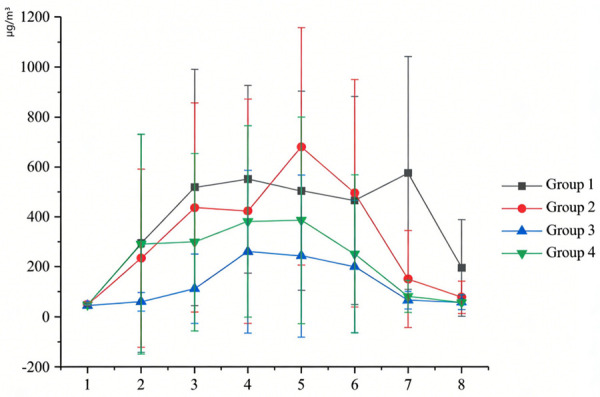
Average PM2.5 concentrations across eight detection times in four groups (Group 1 is traditional suction tube, Group 2 is the 1.5-mm-diameter suction tube, Group 3 is the 2.0-mm-diameter tube, and Group 4 is the 2.5-mm-diameter tube).

Figure 4 illustrates the variation in the concentration of particulate matter at different detection times for the four groups. The concentrations of 2.5 µm particulate matter reached a peak at 15–20 s, and then decreased. The concentration of PM2.5 for the new suction tube with 2.0 mm orifice diameter was significantly lower than the other new schemes at the detection time of 10 s after completion of cutting (*P* < 0.05, Table 3). Meanwhile, the results showed that PM2.5 levels increased slightly when measured at 2 m from the surgeon (*P* < 0.05, Table 4).

**Table 3:** Average concentration of PM2.5 40 cm from the surgeon $\left(\bar{x}\pm \mathrm{SD}\;\mu g/m^{3}\right)$.

Time	Traditional suction tube	Diameter 1.5 mm	Diameter 2.0 mm	Diameter 2.5 mm
Before cut	46.88 ± 5.24	48.75 ± 6.18	44.63 ± 4.98	48.25 ± 1.83
Cut 5 s	294.75 ± 437.81	234.75 ± 356.67	59.63 ± 37.28	291 ± 439.47
Cut 10 s	518.25 ± 473.21	437.13 ± 418.75	112.12 ± 138.63	299.13 ± 355.50
Cut 15 s	551.38 ± 376.40	423.12 ± 449.59	260.38 ± 325.91	382.5 ± 383.33
Cut 20 s	504.75 ± 398.67	682 ± 475.17	243.75 ± 324.99	386.5 ± 413.90
Finish cut 5 s	465.75 ± 416.63	494.75 ± 455.14	200.5 ± 264.46	251.75 ± 316.93
Finish cut 10 s	575.63 ± 466.98	151.38 ± 193.94	(65.88 ± 35.28)^a^	81.5 ± 63.41
Finish cut 15 s	195.5 ± 193.07	77.38 ± 64.64	57.87 ± 28.72	55.88 ± 11.34

^a^Indicates that 10 seconds after electrosurgical cutting, the PM2.5 concentration of the diameter 2.0 mm group was significantly lower than that in the 1.5 mm and 2.5 mm groups (*P* < 0.05).

**Table 4:** Average concentration of PM2.5 2 m from the surgeon $\left(\bar{x}\pm \mathrm{SD}\;\mu g/m^{3}\right)$.

Time	Traditional suction tube	Diameter 1.5 mm	Diameter 2.0 mm	Diameter 2.5 mm
Before cut	57 ± 4.84	59.38 ± 4.65	59.88 ± 9.77	59.13 ± 4.29
Cut 5 s	57 ± 5.52	59.38 ± 4.17	61.38 ± 11.23	65.25 ± 17.39
Cut 10 s	57.25 ± 6.88	59 ± 5.01	59.13 ± 9.03	62.63 ± 13.38
Cut 15 s	57.25 ± 4.92	59.37 ± 4.98	60 ± 9.53	64.38 ± 15.91
Cut 20 s	56.38 ± 4.98	60.25 ± 4.80	59.5 ± 10.37	64.25 ± 16.60
Finish cut 5 s	57.25 ± 5.06	58.88 ± 4.67	59 ± 8.91	64.13 ± 17.19
Finish cut 10 s	57.13 ± 5.30	58.88 ± 4.94	58.63 ± 7.76	63.38 ± 15.74
Finish cut 15 s	58.5 ± 6.82	59.13 ± 5.08	59 ± 7.85	62.13 ± 12.58

### Comparing the three new smoke evacuators

Compared with the traditional suction tube group, the average smoke reduction rates were 24.20% for the 1.5 mm design, 66.86% for the 2.0 mm design, and 43.02% for the 2.5 mm design (Table 5). The design with the orifice diameter of 2.0 mm demonstrated the best smoke evacuation performance.

**Table 5:** Average smoke reduction rate of the three new suction tubes.

Groups	Average concentration of PM2.5 $\left(\bar{x}\pm \mathrm{SD}\;\mu g/m^{3}\right)$	Reduction rate (%)
Traditional suction tube	394.11 ± 209.34	Blank group
Diameter 1.5 mm	318.67 ± 248.42	24.20
Diameter 2.0 mm	130.59 ± 120.22	66.86
Diameter 2.5 mm	224.56 ± 207.71	43.02

## Discussion

The potential hazards of surgical smoke have attracted increasing attention among medical personnel, particularly during the COVID-19 pandemic (23–25). Insoluble fine particulate matter ≤2.5 μm in diameter can reach the alveolar region of the lungs, where clearance occurs through phagocytosis by alveolar macrophages, which may induce inflammatory and pro-thrombotic responses (1, 21). Between January and April 2020, a total of eight US states (Oregon, Utah, Iowa, Illinois, Kentucky, Tennessee, Georgia, and Connecticut) introduced legislation on surgical smoke evacuation, although none of the bills were passed (26). Previous studies have suggested that the suction device be kept within 5 cm of the surgical site in order to effectively remove smoke (27). In view of this, the present study developed a new smoke evacuation instrument to promote widespread adoption of local smoke evacuation and reduce potential hazards to medical staff. To get closer to the incision, the smoke tube was nested outside the conventional suction tube. The suction mechanism of the inner and outer tubes was kept separate. The outer tube wall was perforated to increase the suction area. The newly developed suction instrument allows surgical assistants to simultaneously remove the surgical smoke and suction liquids with one hand. Furthermore, the new instrument is made of polyvinyl chloride, a material with chemical stability, good biocompatibility, and low cost.

This study compared three design schemes to identify the best smoke evacuator. The smoke capture efficiency of the 2.0-mm-diameter suction tube was 66.86%, while the other two schemes were significantly worse, consistent with previous studies (13, 14). In addition, PM2.5 concentration analysis 10 s after completion of cutting revealed that there are statistically significant differences between the traditional suction tube and the 2.0-mm-diameter suction tube. The new instrument with an orifice diameter of 1.5 mm demonstrated the lowest clearance rate of PM2.5, likely due to the small aperture of the pipe. When significant amounts of smoke were produced, the pipe could not suction effectively, allowing smoke to diffuse into the air.

Previous research has shown that when an electrosurgical knife cuts for 3–5 s, particle monitors located 5 cm away from the surgical incision at a height of 10 cm detect an instantaneous increase in particles with a diameter of 3.0 μm (28). In this study, after 5 s of cutting, the average smoke level for the 2.0 mm suction tube remained at a normal level, with smoke concentrations rising slowly during the overall cutting process and immediately declining after the cutting is completed. The overall smoke removal rate of the 2.5 mm design was lower than that of the 2.0 mm design. We also reached the same conclusion when using COMSOL for analysis. The negative pressure at holes 1–4 hole of the 2 mm was greater than that of the 2.5 mm tube. In the COMSOL analysis, the outlet condition of the outer tube was set to a fixed pressure difference of negative 50 Pa, and backflow was suppressed. Results showed that pressures across the individual holes were not the same. We analyzed pressures at each hole in a column of eight holes. For the 2.0 mm outer tube, counting from the front end to the back, the negative pressures for holes 1–4 were 7.05520, 0.52185, 0.04300, and 0.00094 Pa, respectively, whereas for the 2.5 mm tube, they were 6.00930, 0.28681, 0.00710, and 0.00093 Pa, respectively (20). This phenomenon may be related to the large opening area of the 2.5 mm-diameter device. Limited negative pressure from the smoke evacuation system cannot fully suction each orifice, leading to ineffective operation of some openings. Thus, total suction performance does not rise as the opening area increases. In contrast, the 2.0 mm design was better suited for delicate surgical regions, with concentrated negative pressure and stable airflow paths, allowing efficient capture at the source of smoke generation. Therefore, the suction tube with a 2.0 mm orifice diameter was considered to be the optimal scheme.

Vertical laminar flow operating rooms employ a top-down directional airflow, which effectively dilutes, displaces, and swiftly removes contaminants produced in the surgical area. This principle aligns with established clean operating room aerodynamics. Compared with the non-laminar environment examined in this study, vertical laminar flow conditions significantly reduce the diffusion distance and range of surgical smoke in the surrounding space. In addition, the overall concentration of PM2.5 in the air is diminished, and its residence time is shortened. The actual removal efficiency of purification equipment may be further enhanced due to the synergistic effects of the airflow. Consequently, the reduction in PM2.5 observed in this study under non-laminar conditions can be considered a relatively conservative assessment. In a functional laminar flow operating room, the purification effect is unlikely to diminish and may, in fact, improve.

The proposed design is innovative and functions in the same manner as the smoke-evacuating electrosurgical pencil. During the experiment, we observed that it was very difficult for the smoke suction tube to remove all surgical smoke, because when smoke is generated it quickly spreads in the air, due to the limited suction force of negative pressure. The nested smoke removal tube developed in this study costs only 2% of the price of the smoke removal electrocautery pen, yet it achieves a comparable smoke removal effect. Moreover, the surgical smoke is aspirated by the assistant surgeon, thereby minimizing the impact on the primary surgeon. For refinement of the design, discussions were held with surgeons and professional medical device companies. These findings may be popularized and generalized for future research.

## Limitations

The sample size in this study was small, and the experiment was conducted on animals. However, before the animal experiment, a simulation was carried out using COMSOL Multiphysics. Results showed that the 2.5-mm-diameter scheme was the best one, though the difference compared with the 2.0-mm-diameter design was not statistically significant. All the tests were performed in a “laboratory setting” and in an OR without a ventilation system or vertical laminar flow. Furthermore, to ensure data quality, the same pig was used throughout the study, due to which the freshness of pig tissues in the later experiments may have differed from that in earlier ones. Given the various sources of noise in the operating room (ECG monitors, communication among surgeons, etc.), this study was conducted on a deceased pig, and noise levels were not compared. However, noise levels were compared in subsequent clinical trial protocols.

## Conclusion

A new all-in-one suction instrument has been developed to enable simultaneous suction of blood and surgical smoke with one hand, offering advantages compared with currently available smoke evacuation systems. This new tool combines a large smoke suction area, low-cost consumables, and convenient operation.

The aim of this study was to identify the optimal design scheme and provide preliminary evidence regarding the feasibility and usefulness of this new tool. Findings suggest that the 2.0-mm-diameter design is the best one. However, this new tool still requires evaluation in a real clinical setting. This device may serve as a starting point or reference for promoting widespread adoption of local smoke evacuation worldwide.

In the future, we will continue to improve and optimize the design by reducing the diameter of the openings, increasing the number of openings, and comparing the smoke absorption efficiency with the current design.

## Data Availability

The original contributions presented in the study are included in the article/Supplementary Material; further inquiries can be directed to the corresponding author.
